# Allelopathic, Phytotoxic, and Insecticidal Effects of *Thymus proximus* Serg. Essential Oil and Its Major Constituents

**DOI:** 10.3389/fpls.2021.689875

**Published:** 2021-06-15

**Authors:** Shixing Zhou, Caixia Han, Chenpeng Zhang, Nigora Kuchkarova, Caixia Wei, Chi Zhang, Hua Shao

**Affiliations:** ^1^State Key Laboratory of Desert and Oasis Ecology, Xinjiang Institute of Ecology and Geography, Chinese Academy of Sciences, Ürümqi, China; ^2^University of Chinese Academy of Sciences, Beijing, China; ^3^Research Center for Ecology and Environment of Central Asia, Xinjiang Institute of Ecology and Geography, Chinese Academy of Sciences, Ürümqi, China; ^4^Shandong Provincial Key Laboratory of Water and Soil Conservation and Environmental Protection, College of Resources and Environment, Linyi University, Linyi, China

**Keywords:** phytotoxicity, biopesticides, carvacrol, P-cymene, γ-terpinene

## Abstract

The chemical profile of *Thymus proximus* essential oil (EO) and its allelopathic, phytotoxic, and insecticidal activity was evaluated. Carvacrol, p-cymene, and γ-terpinene were detected as the major components of the EO, representing 85.9% of the total oil. About 50 g fresh plant material of *T. proximus* in a 1.5-L air tight container completely inhibited the seed germination of *Amaranthus retroflexus* and *Poa anuua*. Meanwhile, the EO exhibited potent phytotoxic activity, which resulted in 100% germination failure of both the test species when 2 mg/ml (for *A. retroflexus*) and 5 mg/ml (for *Poa annua*) oil was applied. The EO also triggered a significant insecticidal activity on *Aphis gossypii* with a LC_50_ value of 6.34 ppm. Carvacrol was identified as the main active compound responsible for both the plant suppressing effect and the insecticidal activity of the EO. Our study is the first on the allelopathic, phytotoxic, and insecticidal activity of *T. proximus* EO, and the determination of the responsible compound, which indicated their potential of being further explored as environment friendly biopesticides.

## Introduction

Essential oils (EOs) are mixtures of plant-derived secondary metabolites that are extensively applied in food preservation and medical practices for thousands of years (Majewska et al., 2019; Suteu et al., 2020; Giunti et al., 2021). Many aromatic plants are known for their extraordinary ability to produce a large amount of EOs that can repel grazers, kill pests, or inhibit the growth of competing plants growing in the neighborhood (Willmer et al., 2009; Aungtikun et al., 2021; Han et al., 2021; Sousa et al., 2021). Due to these qualities, certain EOs obtained from aromatic plants, including their major constituents, have the potential to be used as environmentally compatible alternatives to synthetic pesticides and herbicides. Successful commercialized examples include clove oil, which is the main active ingredient in the herbicide Burnout II (Bonide Products Inc., Oriskany, NY, USA), and cinmethylin, the phytotoxin 1,4-cineole's derivative that can be detected in EOs of many plants (Grayson et al., 1987; Ahuja et al., 2015). On the other hand, the commercial production of pest management products based on plant EOs appears to have lagged significantly behind, indicating a major disconnect between academic research and industrial practice (Isman, 2017). However, there are some commercial pesticides that contain plant EOs. For example, a commercial product named as “Rice Weevil Eradication” (manufacturer: Hub Club, Siheung, Korea) containing cinnamon [*Cinnamomum cassia* (L.) J. Presl] oil as its active ingredient (Yang et al., 2020). Ecotrol Plus, the flagship agricultural product produced and marketed by KeyPlex Co. (Winter Park, FL, USA), introduced in 2003, contains 10% rosemary oil, 2% peppermint oil, and 5% geraniol as active ingredients.

Worldwide, synthetic chemicals are used in agriculture; however, the extensive application has triggered resistance in pests, not to mention that they can cause many problems not only to the environment but also to human health. Compared to synthetic chemicals, plant-derived natural compounds have the advantages of fast biodegradability, low risk for subsequent pest/weed resistance, and relatively weak toxicity to non-target organisms (Chandler et al., 2011; Isman, 2015; Pavela and Benelli, 2016).

To the best of our knowledge, environment friendly agricultural chemicals are particularly important to drylands, which are characteristic for low precipitation and simple, fragile soil microbiota, which almost unavoidably cause slow degradation rate of synthetic chemicals; the accumulation of synthetic compounds subsequently might result in acute and chronic toxicity to human and herds and pose threat on the environment such as suppressing the growth of desert plants, which lead to increased wind soil erosion (Pavela, 2015). Due to the occurrence of resistance of agricultural pests to synthetic chemicals, farmers have to either increase the amount of application or switch to a new type of pesticides and herbicides, which may considerably increase the costs of maintaining dryland farms (Benhalima et al., 2004; Ahmad and Jaiswal, 2015). In addition, dryland harbors very rich natural resource of medicinal aromatic plants. Many desert aromatic plants belonging to the genus *Thymus* are known for their outstanding ability to produce high productivity and quality of EOs, which have been widely used in pharmaceutical, food, and cosmetic applications (Stahl-Biskup and Saez, 2002; Imelouane et al., 2009). *Thymus proximus* Serg, for example, a dense and robust shrub distributes over a wide range of mountainous regions and predominantly scatters in northwest China and Central Asia dryland (Wu et al., 1983), was found to have antimicrobial, antioxidant, and other biological activities (Jia et al., 2010). *T. proximus* is known for its high productivity of EOs, and like reports on some other desert aromatic plants including *Thymus* species growing in the drylands, its EO might have the allelopathic effect that can either act directly as volatile allelochemicals or accumulate in the soil to impact the growth of neighboring plants (Barney et al., 2009; Inderjit et al., 2011; Ali et al., 2014, 2015; Alexa et al., 2018; Vaiciulyte and Loziene, 2020).

Although some biological activities, such as antimicrobial activity of *T. proximus* EO, have been reported before, its allelopathic, phytotoxic, and insecticidal activities are not studied, and the bioactive compound(s) remains unclear. The objectives of our study include: (i) evaluation of the phytochemical profile of the EO produced by the desert plant *T. proximus* growing in Xinjiang province of China; (ii) assessment of the allelopathic, phytotoxic, and pesticidal effects of the EO and its major components; and (iii) determination of the major active component responsible for the biological activities of the EO.

## Materials and Methods

### Plant Material

Aboveground *T. proximus* Serg. material (flowering shoot) was collected in Tianshan mountains (Lat 43.4268°N, Lon 87.1764°E, with an elevation of 2,006 m) in Xinjiang Province, China in June, 2019. Specimens were identified by Professor Li Wenjun, and a voucher specimen (XJBI018367) was deposited at the Xinjiang Institute of Ecology and Geography, Chinese Academy of Sciences Ownbey Herbarium.

### Extraction of the EO

About 200 g of fresh materials of *T. proximus* was hydrodistillated for 4 h using a Clevenger-type apparatus to extract the EO, and this procedure was repeated three times (altogether 600 g plant material was used) to yield enough oil for the gas chromatography/mass spectroscopy (GC/MS) analysis and the following bioassay. The oil was then dried using anhydrous Na_2_SO_4_ and kept at 4°C.

### GC/MS Analysis

The GC/MS analysis was performed to determine the chemical profile of *T. proximus* EO using a 7890A/5975C GC/MS system (Agilent Technologies, Palo Alto, CA, USA) equipped with a (5%-phenyl)-methylpolysiloxane phase column (30 m × 0.25 mm; film thickness 0.25 μm), DB-5MS (Agilent J&W Scientific, Folsom, CA, USA). The experimental conditions were programmed as follows: Helium (carrier gas) at a flow rate of 1 ml/min; the oven temperature was first held at 50°C for 10 min and then programmed from 50 to 120°C at a rate of 1.5°C/min and from 120 to 240°C at 20°C/min and then held for 5 min; injector and detector temperature: 280°C; sample volume: 0.1 μl; split ratio: 50:1; mass spectra: 70 eV, mass range: *m*/*z* 40–800 amu. Identification of the compounds was determined by comparison of their mass spectra and retention indices (RIs), which were determined by the linear interpolation relative to retention times of a standard mixture of C_7_-C_40_ *n*-alkanes with the data given in National Institute of Standards and Technology (NIST) and published literature (Oladipupo and Adebola, 2009; Shao et al., 2018).

### Allelopathic Effect

Fresh stems and leaves of *T. proximus* were arranged into plastic containers (13.5 × 13.5 × 8.5 cm, volume 1.5 L) at the following ratios: 0 g, 6.67 g, 13.33 g, and 26.67 g/L containers. Their allelopathic potential was assessed by performing bioassays against *Amaranthus retroflexus* L. and *Poa annua* L., which grow in the same habitat alongside *T. proximus*. Seeds of receiver species were surface sterilized with 2% sodium hypochlorite before use. Distilled H_2_O (5 ml) was added to each Petri dish (ϕ 9 cm, lined with a layer of filter paper), followed by sowing of 20 seeds. Each container received one Petri dish that was placed onto the plant material. Containers without plant materials (0 g) were used as the control. All containers were kept open for 5 min each day to allow in the fresh air. *A. retroflexus* and *P. annua* seedlings were measured after 5 and 7 days of incubation, respectively, due to relatively slow development of *P. annua* seedlings. Three replicates were prepared for the bioassay and in total 50 seedlings were measured (Williamson and Richardson, 1988; Wei et al., 2019; *n* = 50).

### Phytotoxic Effect of the EO and Its Major Components

*Amaranthus retroflexus* and *P. annua* were used to evaluate the phytotoxic activity of the EO and its major ingredients. p-Cymene, γ-terpinene, and carvacrol (purity 98%) were purchased from Sigma-Aldrich Co. (St. Louis, MO, USA). Seeds of the test species were surface sterilized with 2% sodium hypochlorite before application of the oil and its major components. *T. proximus* oil and the major components were first dissolved in dimethyl sulfoxide (DMSO, 0.1% v/v final concentration) and then diluted with the distilled water containing Tween 80 (final concentration 0.02%) to yield solutions at 0.25, 0.5, 1, 2, 5, and 10 mg/ml for the assay. Previously, DMSO has been adopted in similar bioassays due to the fact that essential oils and oil constituents are soluble in it, and that DMSO does not pose significant inhibitory effect on test plants (Tanveer et al., 2012; Pinto et al., 2015). The mixture of the three major constituents was prepared by combining p-cymene, γ-terpinene, and carvacrol at the ratio of 44.3:33.2:8.5, which was identical to their relative percentage in the EO to test their possible synergistic/antagonistic effect.

About 5 ml of solutions were added to each Petri dish (ϕ 9 cm; controls received 5 ml of distilled H_2_O containing 0.1% DMSO and 0.02% Tween 80), followed by sowing of 10 test seeds. Petri dishes were sealed with parafilm and kept in a growth cabinet at 25°C with a photoperiod L:D = 16:8. *A. retroflexus* and *P. annua s*eedlings were checked and measured after 5 and 7 days of incubation, respectively, due to relative slow development of *P. annua* seedlings. Five replicates were performed for the assays, and in total 50 seedlings were measured (*n* = 50; Shao et al., 2018).

### Insecticidal Activity of the EO

*Thymus proximus* oil, p-cymene, γ-terpinene, carvacrol and their mixture (ratio of 44.3:33.2:8.5, the relative percentage in the EO) at 2.5, 5, 10, 20, 50, and 100 ppm was impregnated into the Whatman No.2 filter paper (Maidstone, Kent, United Kingdom) discs (1 × 1 cm), which were then taped onto the inner side of the lid of each Petri dishes (9 cm in diameter) to avoid direct contact between the EO/major components and *Aphis gossypii* Glover. Thirty adults of *A. gossypii* were placed onto a healthy fresh black nightshade (*Solanum nigrum*) leaf on a layer of moist filter paper. All Petri dishes were covered and kept in an incubator (25 ± 2°C, photoperiod L:D = 16:8) for 2 days. Mortalities of the adults were determined at 24-h intervals after treatment. Three replicates were performed to measure the insecticidal activity, which was expressed as percent mean mortalities of the adult *A. gossip* (Laborda et al., 2013; Zhou et al., 2019).

### Statistical Analyses

The bioassay experiment followed a completely randomized design with five replications and 50 seedlings for each treatment. Results were expressed as mean ± SE of the mean. One-way ANOVA (*p* < 0.05) was applied using the IBM SPSS statistical package version 21.0 (IBM SPSS, Armonk, NY, USA) for Windows to examine whether the difference of the allelopathic, phytotoxic, and insecticidal effects of the EO produced by *T. proximus*, their major constituents, that is, p-cymene, γ-terpinene, and carvacrol and their mixture tested at different concentrations was significant; then all data were further processed using the Fisher's least significant difference (LSD) test at *p* < 0.05 level to compare the difference among treatments. The inhibitory concentration required for 50% inhibition (IC_50_/LC_50_) values were calculated using the PROBIT analysis (SAS/STAT User's Guide; SAS Institute Inc., Cary, NC, USA).

## Results

### Essential Oil Yield and Composition

The EO of *T. proximus* was obtained by the traditional hydrodistillation method using fresh aboveground plant materials. The yield was 0.35% (v/w, volume/fresh weight). Eventually, 18 compounds were determined, which accounted for 98.51% of the total oil, whereas 1.49% of the oil remained unclassified. The most abundant components were p-cymene (44.26%), γ-terpinene (33.17%), and carvacrol (8.47%), which represented 85.9% of the total oil. Monoterpene hydrocarbons accounted for 86.60% of the total oil, whereas oxygenated monoterpenes and sesquiterpene hydrocarbons represented 10.05 and 1.86% of the total oil, respectively (Table 1).

**Table 1:** Chemical composition of *Thymus proximus* essential oil.

**Compounds**	**RI^**a**^**	**RI^**b**^**	**Area (%)**	**Identification**
α-Thujene	913	924	1.76	MS, RI
α-Pinene	928	938*	0.93	MS, RI
(-)-Camphene	946	952	0.66	MS, RI
4-Thujene	965	969	0.3	MS, RI
β-Pinene	976	982	1.41	MS, RI
δ-Carene	1,006	1,004	2.52	MS, RI
p-Cymene	1,011	1,012	44.26	MS, RI
1,5-Dimethyl cyclooctadiene	1,018	1,017	0.49	MS, RI
β-(Z)-Ocimene	1,038	1,041	0.63	MS, RI
γ-Terpinene	1,047	1,056	33.17	MS, RI
Terpinolene	1,050	1,084	0.47	MS, RI
Borneol	1,142	1,173	0.61	MS, RI
Carvacrol	1,273	1,287	8.47	MS, RI
2-Ethyl-4,5-dimethylphenol	1,281	1,300	0.53	MS, RI
Thymol	1,284	1,287	0.05	MS, RI
Durenol	1,324	1,319	0.39	MS, RI
Caryophyllene	1,401	1,415	1.08	MS, RI
β-Bisabolene	1,495	1,489	0.78	MS, RI
Monoterpene hydrocarbons			86.6	
Oxygenated monoterpenes			10.05	
Sesquiterpene hydrocarbons			1.86	
Total identified			98.51	

*RI^a^, Retention index measured relative to n-alkanes (C_7_-C_40_) using a DB-5MS column; RI^b^, Retention index from literature; MS, mass spectra*.

### Allelopathic Potential

**Figure 1:**
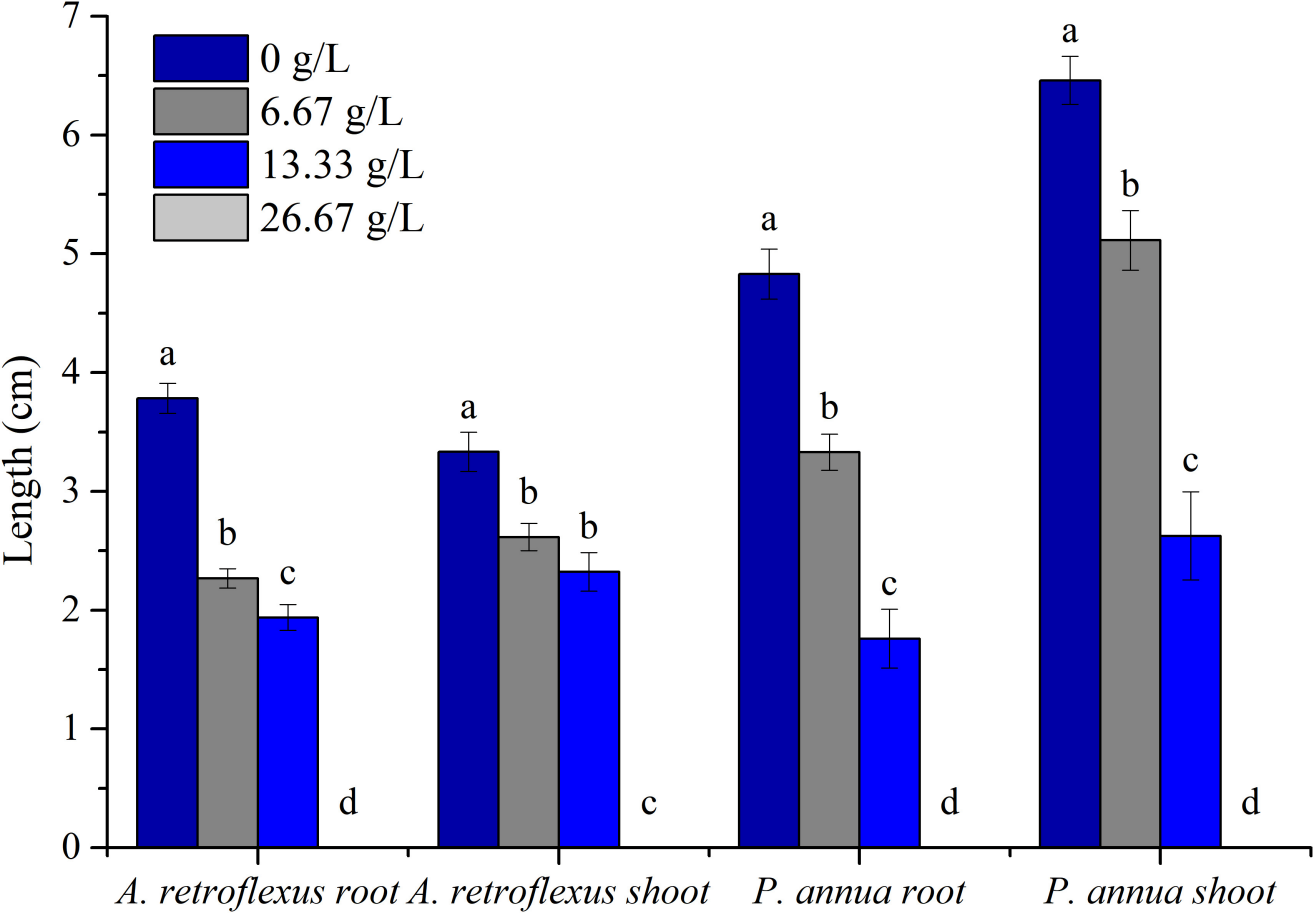
Allelopathic effect of volatile organic compounds (VOCs) released by *Thymus proximus* on the root and shoot elongation of *Amaranthus retroflexus* and *Poa annua*. Means with different letters indicate significant differences at *p* < 0.05 level according to the Fisher's least significant difference (LSD) test.

The allelopathic effect of volatile organic compounds (VOCs) released by *T. proximus* was investigated by arranging fresh aboveground plant parts into air-tight plastic containers; *A. retroflexus* (dicot) and *P. anuua* (monocot), which are found growing in the same habitat alongside *T. proximus*, were selected as the test species VOCs released by *T. proximus* at 6.67 g/L containers suppressed radical elongation of *A. retroflexus* and *P. anuua* by 40.1 and 31.1%, respectively, and 13.33 g/L treatment resulted in the reduction of the root elongation by 48.8 and 63.6% for *A. retroflexus* and *P. anuua*, respectively. About 26.67 g/L treatment basically prohibited the seed germination of two test species. *P. annua* (monocot) was apparently more sensitive compared to *A. retroflexus* (dicot); the IC_50_ values were 14.69 and 14.42 g for *A. retroflexus* and *P. annua* roots, and 20.595 and 16.316 g for shoots, respectively (Figure 1).

### Phytotoxic Activity Bioassay

**Figure 2:**
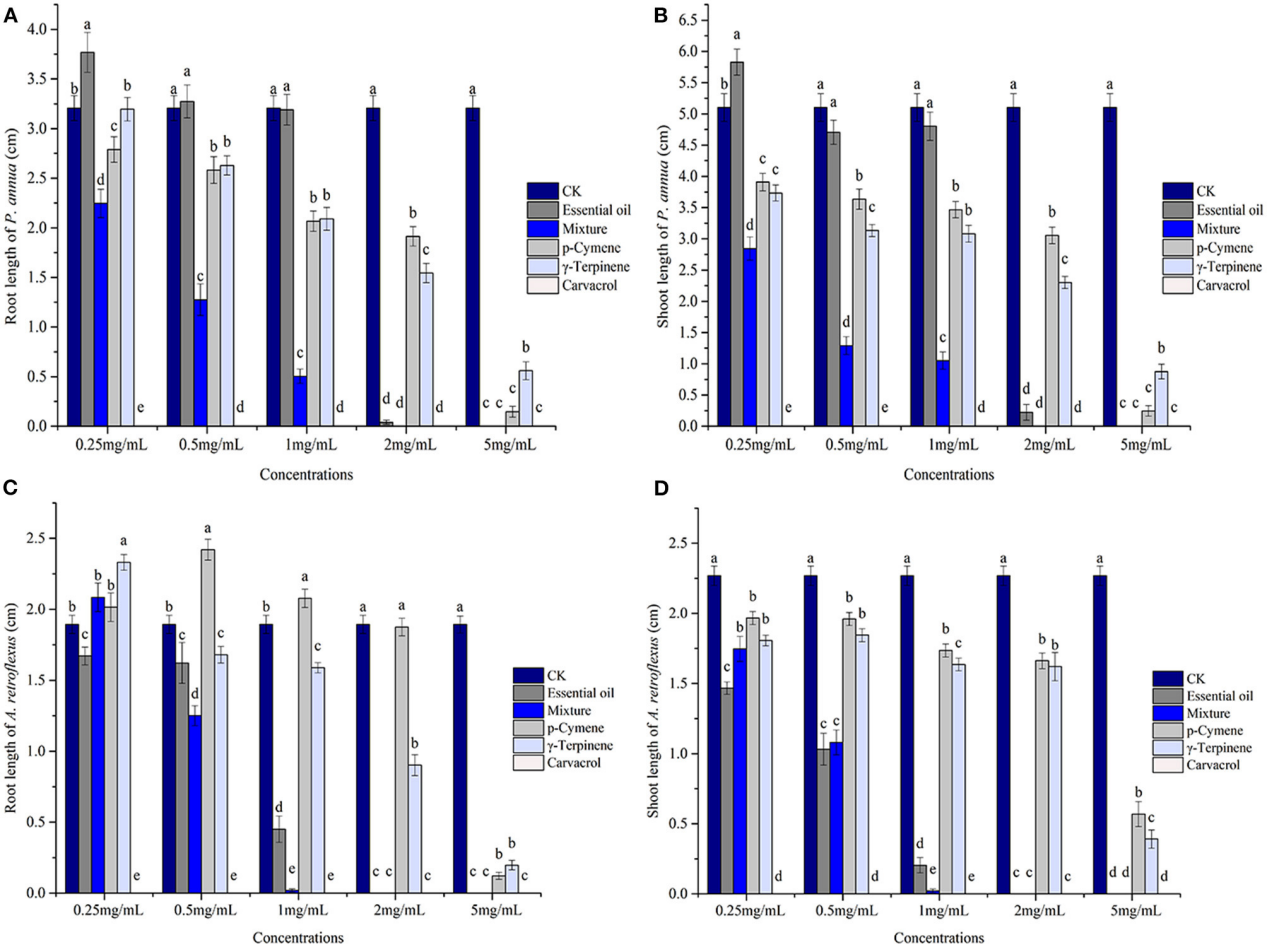
Phytotoxic effects of *Thymus proximus* essential oil (EO) and its major constituents, p-cymene, γ-terpinene, carvacrol, and their mixture on the seedling growth of *Poa annua* and *Amaranthus retroflexus* (*n* = 50). Different letters represent a significant difference at *p* < 0.05 level according to the Fisher's LSD test. **(A)** root length of *P. annua*; **(B)** shoot length of *P. annua*; **(C)** root length of *A. retroflexus*; and **(D)** shoot length of *A. retroflexus*.

Phytotoxic activity of the EO (concentrations applied ranging from 0.25 to 5 mg/ml) and its major components was assessed by comparing their plant regulatory effect on the seedling growth of *A. retroflexus* and *P. annua*. For *A. retroflexus*, p-cymene promoted the root development of *A. retroflexus* at 0.5 mg/ml; however, the inhibitory activity was observed with the increase of concentration, and 5 mg/ml treatment resulted in 93.54% reduction on the root development. γ-Terpinene exhibited relatively stronger activity against *A. retroflexus*, inhibiting the root length by 52.35% at 2 mg/ml, and 89.55% at 5 mg/ml. The third major constituent, carvacrol, showed remarkably stronger activity compared with the other two compounds, which completely suppressed the seed germination at the lowest concentration tested (0.25 mg/ml). The mixture of these three major components exhibited stronger activity than p-cymene and γ-terpinene but much weaker activity than carvacrol, which reduced the root length by 33.92% at 0.5 mg/ml, and 98.94% at 1 mg/ml. When the concentration reached 2 mg/ml, the seed development was completely prohibited. In conclusion, the EO exerted more potent activity than p-cymene and γ-terpinene but much weaker activity than carvacrol; the strength of the EO was comparable but still somewhat weaker than the mixture; the IC_50_ values of p-cymene, γ-terpinene, mixture, and the EO were 4.52, 3.78, 2.06, and 2.60 mg/ml, respectively (Figure 2, Table 2).

**Table 2:** Regression analyses of the phytotoxic effect of *Thymus proximus* essential oil (EO), its major constituents p-cymene, γ-terpinene, and carvacrol, and their mixture on the root and shoot growth of *Amaranthus retroflexus* and *Poa annua*.

**Test plants**	**EO/major components**	**Regression equation**	***r*^**2**^**	**IC_**50**_ (mg/ml)**	**95% CL**
*A. retroflexus* root	p-Cymene	y = 15.753x^2^−71.642x + 51.737	0.951	4.52	3.93–5.11
	γ-Terpinene	y = 26.643x−50.704	0.958	3.78	3.25–4.31
	Carvacrol	–	–	–	–
	Mixture	y = −10.857x^2^ + 93.768x−97.324	0.965	2.06	1.48–2.64
	Essential oil	y = 26.221x−18.216	0.873	2.60	2.05–3.15
*A. retroflexus* shoot	p-Cymene	y = 6.3663x^2^−24.564x + 34.084	0.927	3.03	2.71–3.35
	γ-Terpinene	y = 7.3667x^2^−30.733x + 46.836	0.903	4.27	3.94–4.60
	Carvacrol	–	–	–	–
	Mixture	y = −7.4577x^2^ + 64.903x−37.786	0.961	1.67	1.23–2.11
	Essential oil	y = −4.7013x^2^ + 45.681x−9.1479	0.964	1.54	1.17–1.91
*P. annua* root	p-Cymene	y = 6.143x^2^−18.292x + 28.067	0.938	3.89	3.49–4.29
	γ-Terpinene	y = 19.82x−21.954	0.982	3.63	3.24–4.02
	Carvacrol	–	–	–	–
	Mixture	y = 17.99x + 20.915	0.903	1.62	1.25–1.99
	Essential oil	y = 4.8065x^2^ + 4.751x−31.199	0.853	3.65	2.93–4.37
*P. annua* shoot	p-Cymene	y = 7.4384x^2^−29.138x + 49.486	0.923	3.93	3.57–4.29
	γ-Terpinene	y = 3.335x^2^−7.1789x + 33.397	0.965	3.55	3.28–3.82
	Carvacrol	–	–	–	–
	Mixture	y = 13.68x + 38.628	0.889	0.83	0.55–1.11
	Essential oil	y = 31.638x−55.906	0.865	3.35	2.71–3.99

*r^2^: adjusted coefficient of determination.*

*IC_50_: the inhibitory concentration required for 50% inhibition.*

*95% CL: 95% confidence limits.*

*(–): not calculable*.

Similarly, for the monocot plant *P. annua*, carvacrol exhibited the most potent activity, which completely suppressed its seed germination at 0.25 mg/ml, the lowest concentration applied in the assay. The EO exhibited comparable activity compared with p-cymene and γ-terpinene, whose IC_50_ values were 3.65, 3.89, and 3.63 mg/ml, respectively, and the mixture showed much stronger activity with the IC_50_ value of 1.62 mg/ml (Figure 2, Table 2).

**Figure 3:**
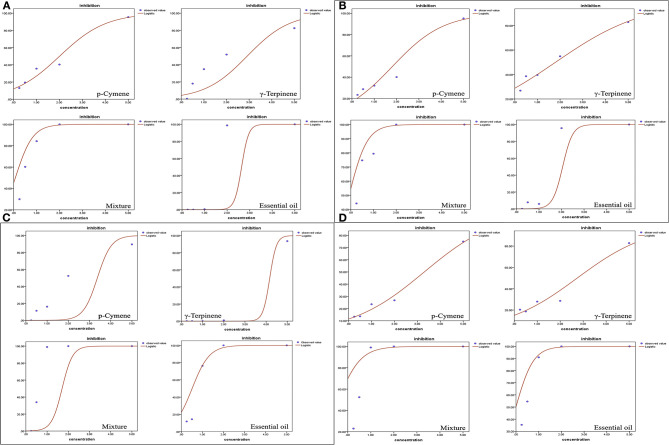
Dose–response curves of *Thymus proximus* essential oil (EO) and its major constituents affected on plant growth of *Poa annua* and *Amaranthus retroflexus*. **(A)** root length of *P. annua*; **(B)** shoot length of *P. annua*; **(C)** root length of *A. retroflexus*; and **(D)** shoot length of *A. retroflexus*.

However, the seedling growth exhibited a similar pattern as the root development to a lesser extent. Carvacrol caused complete failure of the seed development of *P. annua* and *A. retroflexus* at the lowest concentration tested (0.25 mg/ml), whereas the other components and EO started to inhibit shoot growth of *A. retroflexus* at 0.25 mg/ml as well, representing 13.32, 20.37, 23.02, and 35.36% for p-cymene, γ-terpinene, a mixture of main components, and EO, respectively. When the concentration raised to 5 mg/ml, the EO and mixture of major ingredients completely inhibited the shoot growth, while p-cymene and γ-terpinene suppressing the shoot growth of *A. retroflexus* by 74.93 and 82.76%, respectively. The effect of *P. annua* is similar to *A. retroflexus*, with IC_50_ values of 3.93, 3.55, 0.83, and 3.35 mg/ml for p-cymene, γ-terpinene, and mixture of principal components and EO, respectively (Figure 2, Table 2). The dose–response curve of the phytotoxic activity was shown in Figure 3.

### Insecticidal Activity

**Figure 4:**
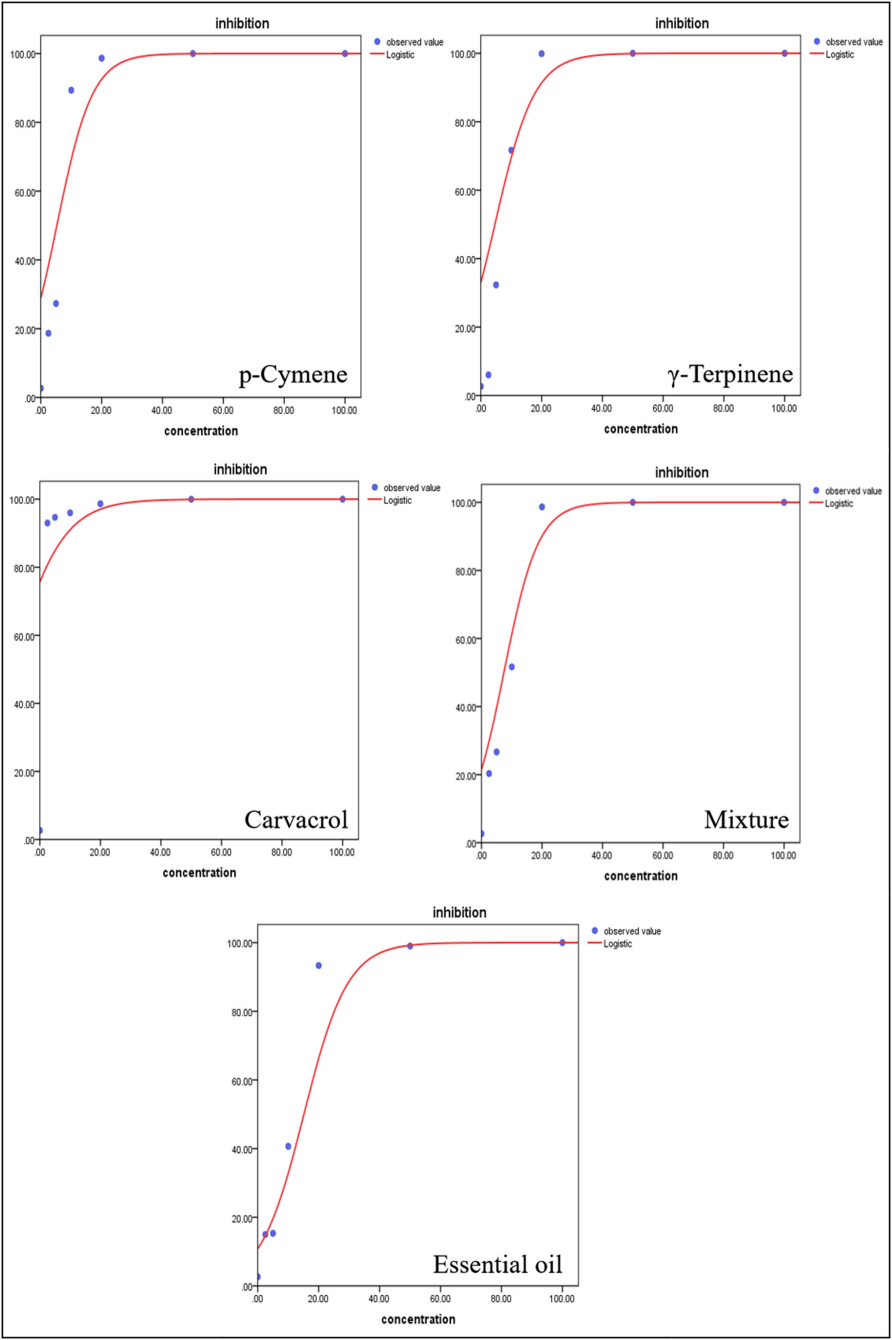
Dose–response curves of *Thymus proximus* essential oil (EO) and its major constituents against *Aphis gossypii* adults.

The insecticidal activity of *T. proximus* EO was determined on adjusted mortality rates of *A. gossypii* at concentrations ranging from 2.5 to 100 ppm. Results showed that *T. proximus* EO had obvious behavioral avoidance and lethal action on *A. gossypii*. The EO, its major components, and their mixture killed all the tested insects at the dose of 100 ppm after 24 h of exposure. The mortality rates of *T. proximus* under 2.5, 5, 10, 20, and 50 ppm, the EO treatments reached 15, 15.33, 40.67, 93.33, and 99.00%, respectively, after 24 h of exposure to the oil. Carvacrol showed the strongest activity against *A. gossypii* with a LC_50_ value of 0.1 ppm, compared with the LC_50_ values of 9.63, 5.69, 6.8, and 7.34 ppm for the EO, p-cymene, γ-terpinene, and the mixture of three major components, respectively (Table 3). The dose–response curve of the pesticidal activity was shown in Figure 4.

**Table 3:** Toxicity of *Thymus proximus* essential oil (EO), p-cymene, γ-terpinene, carvacrol, and their mixture against *Aphis gossypii* adults.

**EO/major components**	**Regression equation**	***r*^**2**^**	**LC_**50**_ (ppm)**	**95% CL**
EO	y = 0.053 + 3.202x	0.922	9.63	4.29–16.63
p-Cymene	y = 0.95 + 3.882x	0.939	5.69	3.75–8.04
γ- Terpinene	y = 0.729 + 4.348x	0.997	6.8	6.16–7.47
Carvacrol	y = 1.94 + 0.963x	0.896	0.1	0.00–0.46
Mixture	y = 0.398 + 2.79x	0.831	7.34	3.81–12.58

*$r_{\text{adj}}^{2}$: adjusted coefficient of determination.*

*LC_50_: 50% lethal concentration of A. gossypii.*

*95% CL: 95% confidence limits*.

## Discussion

A large body of literature has reported the chemical composition of the EOs produced by *Thymus* species, which were frequently found to have abundant thymol, carvacrol, p-cymene, γ-terpinene, caryophyllene oxide, etc. (Kabouche et al., 2005; Hazzit et al., 2006; Sanja and Milka, 2015; Zeynep et al.,, 2018; Behnaz et al., 2020). Marla et al. (2008) investigated the chemical composition of *T. vulgaris* EO and found it was rich in thymol (57.7%), p-cymene (18.7%), and carvacrol (2.8%). Behnaz et al. (2017) evaluated the EOs produced by14 *Thymus* accessions belonging to 10 species and found that their major components were thymol (12.4–79.74%), carvacrol (4.37–42.14%), geraniol (0.3–22.44%), and p-cymene (0.8–12.86%). As of the EO of *T. proximus*, Jia et al. (2010) identified 60 compounds from the EO of *T. proximus*, which accounted for 99% of the total oil, with p-cymene (25.4%), γ-terpinene (18.0%), and thymol (28.0%) being the most abundant components, which was consistent with our results. It is noteworthy to mention that there are various factors that might affect the chemical profile of plant-derived EOs including but not limited to species variety, growth period, geographic locality, surrounding climate, stress, and post-harvest processing, etc. (Raut et al., 2014).

Many plants are capable of synthesizing and releasing VOCs that are found to play key roles in attracting seed-disperser and pollinators, defense against pathogenic fungi and herbivores, interplant signaling, and allelopathic action (Pichersky and Gershenzon, 2002; Dudareva et al., 2013; Adebesin et al., 2017). Wei et al. (2019) reported that VOCs produced by *Atriplex cana* Ledeb. negatively affected the seedling development of *A. retroflexus* and *P. annua*, with 80 g of fresh *A. cana* leaves and stems in a 1.5-L airtight container almost completely prohibited the seed germination of the test plants. Volatiles emitted from the leaves of star anise (*Illicium verum* Hook. f.) totally inhibited the seedling growth of *Lactuca sativa* L., and the major volatile compounds were identified as α-pinene, β-pinene, camphene, 1,8-cineole, D-limonene, camphor, and L-fenchone (Kang et al., 2019). Tang et al. (2019) reported the allelopathic activity of VOCs released by the exotic invasive weed *Xanthium sibiricum* and found that at 80 g fresh plant materials in a 1.5-L airtight container, root growth of receiver plants *A. retroflexus* and *P. annua* was reduced by 49.1 and 69.6%, respectively. The release of volatile allelochemicals into the surroundings are believed to be able to facilitate the dominance of the donor species; and interestingly, herbivore-infested plants are found to produce volatiles to mediate plant–plant interactions by triggering the expression of volatiles of neighboring unattacked plants to decrease their susceptibility to herbivores (Ruther and Kleier, 2005).

There have been a number of reports on the phytotoxic effects of EOs and their constituents, especially monoterpenes, on the seed germination and seedling growth of the test species (Langenheim, 1994; Vokou et al., 2003; Nishida et al., 2005; Salamci et al., 2007). EOs produced by *Thymus* species have also been studied for their phytotoxicity. Ali et al. (2015) found that EOs obtained from different plant parts of *T. algeriensis* inhibited both the shoot and root growth of *Medicago sativa* L. and *Triticum astivum* L. seedlings at the lowest tested concentration (0.1 mg/ml). *T. daenensis* Celak. EO significantly suppressed the seedling development of *A. retroflexus*, and 600 μl/l oil almost completely prohibited its seedling growth (Kashkooli and Saharkhiz, 2014). Another species of the *Thymus* genus, *T. eigii*, showed significant herbicidal activity against *L. sativa, Lepidium sativum* L., and *Portulaca oleracea* L., with 0.5 mg/ml oil completely suppressed the seed germination of all the tested species (Zeynep et al.,, 2018). Sara et al. (2019) investigated the herbicidal action of *T. fontanesii* EO and found 0.03% *T. fontanesii* oil inhibited the seed germination by 100% on *Sinapis arvensis, Avena fatua, Sonchus oleraceus*, and *Cyperus rotundus*. In another study comparing the strength of phytotoxicity of 12 EOs produced by the Mediterranean aromatic plants conducted by Rolim et al. (2010), thyme, balm, vervain, and caraway EOs were found to be more active on germination and radicle elongation of receiver species; among them, thyme oil completely inhibited the seed germination of *Lepidium sativum, Raphanus sativus* L., and *L. sativa* at 1.25 μg/ml. Synowiec et al. (2017) also performed a study comparing the phytotoxicity of 12 EOs and detected that *T. vulgaris, Carum carvi* L., *Mentha piperita* L., and *Salvia officinalis* L. oils possessed the most potent activity, with the ED_50_ values for thyme oil ranging between 0.06 and 1.03 g/L against seven tested plants, which was comparable to our findings (Synowiec et al., 2017). *Thymus pulegioides* L. EO with high content of α-terpinyl acetate inhibited the seed germination and radicle growth for high economic productivity forage grass monocotyledon *Poa pratensis* L (Vaiciulyte et al., 2021). Different extraction methods also cause differences in EO activity. *Thymus decussatus* EO extracted using hydrodistillation method inhibited the seed germination, shoot growth, and root growth of lettuce by 86.6, 87.4, and 89.9%, respectively, whereas the EO extracted using the microwave-assisted techniques method inhibited *L. sativa* by 77.7, 8 5.8, and 84.6% at 100 μl/L, respectively (Saleh et al., 2020).

The phytotoxic effect of a particular EO can be mainly ascribed to certain toxic component(s). Monoterpene compounds have been reported to show strong inhibitory effects on the seed germination of many crops and weeds (López et al., 2008; Li et al., 2011; Ali et al., 2015). The outstanding phytotoxic activity of thymol has been previously studied. Thammyres et al. (2018) found that thymol exhibited phytotoxicity at different concentrations (0.375–3 mmol/L), as reflected on the reduced germination rate of tested monocot and dicot species. Kordali et al. (2008) found carvacrol and thymol prohibited the seed germination and seedling development of *A. retroflexus, Chenopodium album*, and *Rumex crispus* L., whereas p-cymene did not exert a significant phytotoxic activity (8.6 mg/Petri dishes). Consistent with this study, Vasilakoglou et al. (2013) reported thymol completely restrained the seed germination of rigid ryegrass (*Lolium rigidum* Gaudin) at 160 nl/cm^3^ or above, whereas p-cymene was found to be only slightly phytotoxic. Martino et al. (2010) tested the antigerminative activity of thymol, p-cymene, and γ-terpinene, along with other monoterpenes and found that thymol negatively affected the radicle elongation of garden cress significantly at 10^−3^ M, p-cymene suppressed the root growth of garden cress at 10^−4^ M; however, γ-terpinene did not exert any significant effect at tested concentrations. Meanwhile, the isomer of γ-terpinene, that is, α-terpinene, was detected to be phytotoxic against maize seedlings by reducing the root growth, changing the root border cells number, increasing the pectin methyl esterase activity, and upregulating the repel expression in the roots (Wang et al., 2019). In conclusion, thymol exhibited much stronger phytotoxic activity compared with p-cymene and γ-terpinene, implying its role as the major active compound responsible for phytotoxicity of the oil.

Natural products with plant origin can play crucial roles in pest management practice (Faraone et al., 2015; Barua et al., 2020; Basaid et al., 2020; Chen and Oi, 2020). Previously, EOs synthesized by *Thymus* species have been demonstrated to possess insecticidal activity. *T. serpyllum* L. and *T. vulgaris* EOs presented a LC_50_ of < 10 mg/dm^3^ against the pest (*Acanthoscelides obtectus* say) of kidney bean (*Phaseolus vulgaris* L.) after 24 h of exposure (Regnault-Roger et al., 1993). Isman et al. (2001) tested 21 EOs for their insecticidal action *via* topical administration to third instar larvae of the tobacco cutworm, *Spodoptera litura*, and EOs of *Satureja hortensis, Origanum creticum*, and *T. serpyllum* exhibited over 90% larval mortality at 24 h at 100 μg/larva; the LD_50_ value for *S. hortensis* (48.4 μg) was comparable to that for *T. vulgaris* (46.9 μg). Park et al. (2017) detected that the LC_50_ values of *T. vulgaris* EO against *Pochazia shantungensis* nymphs using the leaf dipping bioassay was recorded as 57.48 and 75.80 mg/L for adults using the spray bioassay method. Pavela (2005) tested the insecticidal activity of *T. mastichina* and *T. vulgaris* EOs against *Spodoptera littoralis* larvae and determined their LD_50_ values were 19.3 and 22.9 ml/m^3^. *Thyme* (*T. vulgaris*) EO was also found to show high activity against *Lycoriella ingenua* at 20 × 10^−3^ mg/ml air (Park et al., 2008). EO produced by *T. satureioides* had moderate toxicity with the LD_50_ value of 0.31 μl/cm^2^ and the LD_90_ of 0.77 μl/cm^2^ against the important stored-product pest insect *Tribolium castaneum* (Kasrati et al., 2015). Ali et al. (2015) found the EOs obtained from all organs of *T. algeriensis* possessed strong insecticidal activity (LC_50_ = 44.25–112.75 μl/L air) against cotton leafworm larvae (*Spodoptera littoralis*). In a recent study, EOs of *T. spinulosus* and *T. longicaulis* were assayed for their insecticidal toxicity, and their LC_50_/LD_50_ values were detected in the range of 39.6–87.1 μg/larva, 21.7–62.4 μl/L, and 35.9–147.3 μg/adult, for *Culex quinquefasciatus, Spodoptera littoralis*, and *Musca domestica*, respectively; it is noteworthy to mention that they found the most active samples were those with the highest amounts of thymol (Pavela et al., 2019). *A. gossypii* were reported to be susceptible to a variety of EOs. For example, the strength of *Santalum austrocaledonicum* Vieill EO was comparable to imidacloprid (a neonicotinoid insecticide) against *A. gossypii* infesting Rose of Sharon (*Hibiscus syriacus* L.) with 98.8% mortality (Roh et al., 2015). In another study, *Melaleuca styphelioides* Sm. EO exhibited strong fumigant toxicity on adults and nymphs of *A. gossypii*; 263.18 μl/L air EO let to 100% mortality of this insect (Albouchi et al., 2018).

Previous works have demonstrated the insecticidal activity of carvacrol, p-cymene, and γ-terpinene; in fact, carvacrol was speculated to be the main insecticidal compound of the EOs (Pavela and Sedlák, 2018; Pavela et al., 2019). Park et al. (2017) measured the insecticidal activity of thymol, carvacrol, citral, 2-isopropylphenol, 3-isopropylphenol, and 4-isopropylphenol against *Pochazia shantungensis* adults, and their LC_50_ values were 28.52, 56.74, 89.12, 71.41, 82.49, and 111.28 mg/L, respectively. Dias et al. (2019) evaluated the toxicity of thymol, cinnamaldehyde, carvacrol, eugenol, and trans-anethole on *Mahanarva spectabilis* eggs, nymphs, and adults, and they found that treatments with eugenol, carvacrol, and thymol showed the highest mortalities, presenting efficiencies higher than 85% after 48 h of application. Traboulsi et al. (2002) found that the compounds thymol, carvacrol, (1R)-(+)-α-pinene, and (1S)-(–)-α-pinene showed potent toxicity (LC_50_ 36–49 mg/L), whereas menthone, 1,8-cineole, linalool, and terpineol (LC_50_ 156–194 mg/L) were less toxic to the mosquito *Culex pipiens* molestus. On the other hand, p-cymene and γ-terpinene also showed effective insecticidal activity. Cetin et al. (2010) detected that γ-terpinene triggered ≥90% knockdown against adult *Hyalomma marginatum* at 105 min through 3 h, meanwhile at 24 h only about 87% of the ticks were dead. Another study found that both γ-terpinene and terpinen-4-ol exhibited a significant insecticidal effect on *Spodoptera littoralis* and *A. fabae*; however γ-terpinene was more toxic than terpinen-4-ol, with the LC_50_/LD_50_ values being 23.94 g/L, 18.03 g/L for γ-terpinene, and 32.94 g/L, 20.77 g/L for terpinen-4-ol, respectively (Abbassy et al., 2009). Silva et al. (2018) tested the activity of p-cymene and γ-terpinene against *Rhipicephalus microplus* and revealed their LC_50_ values were 1.41 and 3.08 mg/ml, respectively. Tak and Isman (2017) tested the insecticidal activity of thymol, p-cymene, and their mixture against *Trichoplusia ni*, and the LC_50_ values were 244.3, 875.4, and 534.8 μg/insect after 24 h of treatment, respectively; they also suggested that p-cymene seemed to enhance penetration of thymol through the integument. There was a study comparing the insecticidal activity of 11 Apiaceae plant EOs and their components on adult male and female *Blattella germanica*, and p-cymene and γ-terpinene were found to exhibit significant fumigant toxicity against adult *Blattella germanica*, whereas p-cymene exerted potent contact toxicity against adult *Blattella germanica* (Yeom et al., 2012). In the case of *T. proximus* EO, we discovered that the insecticidal activity of p-cymene, γ-terpinene, and the mixture of three major constituents was much weaker than carvacrol, which suggested that carvacrol might be the main responsible insecticidal compound in the oil. Moreover, these results supported the speculation that carvacrol was the major insecticidal compound of some EOs (Pavela and Sedlák, 2018; Pavela et al., 2019).

Among the three major constituents, carvacrol was found to possess much stronger biological activity compared with p-cymene and γ-terpinene, although they are similar aromatic monoterpenoids. A recent study revealed that monoterpenoids can induce cell membrane dysfunction and interfere with cell metabolism, and OH^−^- and O^−^-radicals are considered to react with cellular components affecting homeostasis (Scariot et al., 2020). By comparing the chemical structures of carvacrol and p-cymene, it is speculated that the hydroxyl group of carvacrol might be critical for its potent activity. In another report, carvacrol was found to possess herbicidal activity due to its ability to incite membrane leakage (Chaimovitsh et al., 2017). On the other hand, it is noteworthy to mention that it is also possible that minor components in the oil might play important roles in the activity. Furthermore, due to the fact that EOs are composed of small molecules that can easily evaporate in the air, the optimization of the formula is necessary so as to stabilize the oils and their constituents.

## Conclusion

Essential oils are valuable sources of providing candidate compounds as potential environment friendly pesticides and herbicides, which can be utilized in pest and weed control safely due to their ability to degrade in nature, and the fact that they are less toxic to the environment. Our study is the first report on the allelopathic, phytotoxic, and pesticidal activities of the EO extracted from the aromatic plant *T. proximus* and on the determination of the major active compound, that is, carvacrol, to be responsible for the biological activity of the oil, implying their potential value of being explored as pesticides and herbicides. Limitations of our work include that only fumigant method was used to evaluate the insecticidal activity of *T. proximus* EO and its constituents against *A. gossypii*, and leaf dipping method needs to be performed in the future on more insects such as stored product pests.

## Data Availability Statement

The original contributions presented in the study are included in the article/supplementary material, further inquiries can be directed to the corresponding author/s.

## Author Contributions

SZ drafted the manuscript and wrote the manuscript. CH drew the scheme and edited the manuscript. CW performed the literature survey. CheZ performed the correction. NK edited and improved the scheme artwork. ChiZ and HS conceptualized, wrote, and edited the manuscript, and they performed the literature survey and ideation of the scheme. All authors contributed to the article and approved the submitted version.

## Conflict of Interest

The authors declare that the research was conducted in the absence of any commercial or financial relationships that could be construed as a potential conflict of interest.
